# Azithromycin Has Been Flying Off the Shelves: The Italian Lesson Learnt from Improper Use of Antibiotics against COVID-19

**DOI:** 10.3390/medicina58030363

**Published:** 2022-03-01

**Authors:** Pietro Ferrara, Luciana Albano

**Affiliations:** 1 Center for Public Health Research, School of Medicine and Surgery, University of Milan—Bicocca, 20900 Monza, Italy; p.ferrara5@campus.unimib.it; 2 Department of Experimental Medicine, School of Medicine and Surgery, University of Campania “Luigi Vanvitelli”, 80138 Naples, Italy

**Keywords:** azithromycin, COVID-19, inappropriate prescribing, SARS-CoV-2, syndemic, Italy

## Abstract

The warning by the Italian Medicines Agency on the high shortage of azithromycin in the country in January 2022 represents a paradigmatic lesson learnt from improper use of antibiotics during COVID-19 pandemic.

## 1. Foreword

On 13 January 2022, the Italian Medicines Agency (Agenzia Italiana del Farmaco—AIFA) warned about a high shortage of azithromycin in the country, mainly due to its improper use against COVID-19. The difficulty in finding the molecule seems to be related to an excessive prescribing in the last two months, coinciding with the rapid increase in cumulative incidence of SARS-CoV-2 cases sustained by the B.1.1.529 variant (Omicron Variant of Concern) [1].

The COVID-19 pandemic has led to a race towards treatments against the disease. Among the possible medications that have been proposed for improving its prognosis, there is no recommendation for treating COVID-19 with antibiotics. In particular, extensive evidence has demonstrated that azithromycin is not associated with a protective effect against the infection and COVID-19 outcomes, despite being initially suggested as one such possible treatment by virtue of potential interference with SARS-CoV-2 infectivity [2].

In Italy, patients can obtain antibiotics in community or hospital pharmacies under prescription by a doctor. It can be thus inferred that azithromycin hoarding represents a symptom of excessive prescribing by healthcare providers, virtually attributable to convincing solicitation by COVID-19 patients or those who are afraid of being infected with SARS-CoV-2. Indeed, COVID-19 is a perfect storm for *infodemic*, with people constantly updated with fake news [3,4].

The principal outcome of the inappropriate prescribing is the delayed care of other infections, such as bacterial community-acquired pneumonia, acute bacterial exacerbations of chronic obstructive pulmonary disease, non-gonococcal or gonococcal urethritis and cervicitis, and other bacterial infections [3]. For azithromycin users, the main consequence is related to the unnecessary exposure to adverse reactions, which may include gastrointestinal problems, allergic reactions, or abnormal heart rhythm (including a life-threatening fast heart rate).

In the case of antibiotics, organisms’ drug resistance is a key additional concern, one of the biggest challenges to public health and healthcare. In Italy, according to the AIFA National Report on Medicines Use for 2020, antibiotics are among the most commonly prescribed drugs, with more than 20 daily defined doses consumed per 1000 persons per day; around 4 in 10 people have received at least one antibiotic prescription [5,6]. As a consequence, Italy is top in the European Union for antimicrobial resistance and its health and healthcare consequences, including the elevated death toll caused by multi-drug-resistant bacteria and increased healthcare resource utilization and costs [5,6]. Of note, the excessive use of azithromycin has been associated with significant changes in the antimicrobial resistance profile of several bacteria (including the population of human gut microbiota) and is also considered to propagate broad nonmacrolide resistance to other antibiotic classes [7,8]. Thus, it can be speculated that the improper use of azithromycin, as of other antibiotics, against COVID-19 has a relative impact on the health consequences of antimicrobial resistance.

The rapid spread of the COVID-19 pandemic caught off guard healthcare systems worldwide, with enormous impact and disruption of healthcare systems and practices [9]. This also concerned antibiotic prescribing and use, particularly in specific acute and intensive care settings [10]. Indeed, the mass—and, in most cases, inappropriate—antibiotic administration during the last two years has increased the carriage of resistant bacteria and infections [10].

Taken together, risks associated with indiscriminate and improper antibiotic use—exacerbated by the COVID-19 pandemic—call for informative intervention targeting providers, including primary health care physicians and pharmacists, as well as the general public.

Specifically, Health and Public Health Authorities should promote specific initiatives tending to improve awareness and knowledge towards antimicrobial resistance and appropriate antibiotic use. These may include the introduction of mandatory programs in medical degree and trainings; evidence-based initiatives tending to reduce the level of inappropriate prescriptions; and the setting up of data flows to adequately inform stakeholder on antimicrobial agent use and resistance through digital health facilities [11,12].

In a nutshell, while we will continue to struggle with COVID-19 pandemic and its consequences, it is necessary to learn the lesson from the case of azithromycin in Italy and promote urgent global public efforts to tackle an irredeemable *syndemic* of COVID-19 and antimicrobial resistance.

## Data Availability

Not applicable.
